# Intrauterine Growth Restriction and the Fetal Programming of the Hedonic Response to Sweet Taste in Newborn Infants

**DOI:** 10.1155/2012/657379

**Published:** 2012-07-18

**Authors:** Caroline Ayres, Marilyn Agranonik, André Krumel Portella, Françoise Filion, Celeste C. Johnston, Patrícia Pelufo Silveira

**Affiliations:** ^1^Núcleo de Estudos da Saúde da Criança e do Adolescente, Hospital de Clínicas de Porto Alegre, Universidade Federal do Rio Grande do Sul, Rua Ramiro Barcelos, 2350, 90035-903 Porto Alegre, RS, Brazil; ^2^School of Nursing, McGill University, Montreal, QC, Canada 43A 2A7; ^3^Departamento de Pediatria e Puericultura, Faculdade de Medicina, Universidade Federal do Rio Grande do Sul, Ramiro Barcelos, 2350, Largo Eduardo Zaccaro Faraco, 90035-903 Porto Alegre, RS, Brazil

## Abstract

Intrauterine growth restriction is associated with increased risk for adult metabolic syndrome and cardiovascular disease, which seems to be related to altered food preferences in these individuals later in life. In this study, we sought to understand whether intrauterine growth leads to fetal programming of the hedonic responses to sweet. Sixteen 1-day-old preterm infants received 24% sucrose solution or water and the taste reactivity was filmed and analyzed. Spearman correlation demonstrated a positive correlation between fetal growth and the hedonic response to the sweet solution in the first 15 seconds after the offer (*r* = 0.864, *P* = 0.001), without correlation when the solution given is water (*r* = 0.314, *P* = 0.455). In fact, the more intense the intrauterine growth restriction, the lower the frequency of the hedonic response observed. IUGR is strongly correlated with the hedonic response to a sweet solution in the first day of life in preterm infants. This is the first evidence in humans to demonstrate that the hedonic response to sweet taste is programmed very early during the fetal life by the degree of intrauterine growth. The altered hedonic response at birth and subsequent differential food preference may contribute to the increased risk of obesity and related disorders in adulthood in intrauterine growth-restricted individuals.

## 1. Introduction

The fetal origins of adult disease hypothesis states that environmental factors, particularly nutrition, act in early life to program the risks for chronic diseases in adulthood [1]. In particular, intrauterine growth restriction (IUGR) is known to be associated with insulin resistance [2, 3], obesity [4–7], and cardiovascular disease [8, 9] in adult life. 

As eating habits can contribute to the development of obesity, diabetes, and cardiovascular disease, one could suggest that persistent nutrient imbalances across the life-span in people who were born IUGR may explain, at least partially, their increased risk to develop metabolic syndrome later in life. Interestingly, studies from different research groups have shown that IUGR individuals indeed have specific food preferences in adulthood [10–12], naturally choosing to eat more foods rich in carbohydrates and/or fat than non-IUGR individuals. Besides, IUGR girls are more impulsive when facing a sweet reward already at 3 years of age [13]. Therefore, IUGR is associated with changes in feeding behavior and preferences that may promote the metabolic changes previously described in this group.

A possible mechanism by which IUGR could permanently alter an individual's food choices is the programming of the sensitivity to the hedonic signaling (i.e., pleasure) associated with the ingestion of a palatable food. Hedonic sensation is reflected in positive patterns of affective orofacial expressions that are homologous between humans and rodents [14–16]. As explained by Berridge [16], the affective pattern of taste reactivity components reflects palatability or affect more closely than it reflects either ingestion or sensation [17, 18]. It was already shown that prenatal protein malnutrition changes the response to reward in adult rodents [19], which may suggest that the same phenomena may be happening in humans. This group of evidence prompted us to propose the hypothesis that IUGR leads to fetal programming of the hedonic responses to the sweet taste, and in the current study we aimed at verifying if IUGR would be related to an altered pattern of affective orofacial expressions to the sweet taste very early in life, addressing this question in preterm infants in their first day of life.

## 2. Subjects and Methods 

This study was performed by secondary analyses of nonused data collected for the purpose of a different project. The original protocol was developed with the objective to investigate the efficacy of routine sucrose analgesia for procedural pain in the first week of life in preterm infants, and was described in detail elsewhere [20, 21]. Briefly, a level III university-affiliated NICU in Canada was the site for the study, providing ethics approval by a constituted review board. Infants, who were born between 25 and 31 completed weeks' postconceptional age, were expected to live according to the opinion of the attending neonatologist, were above the fifth percentile weight for gestational age, had intraventricular hemorrhage less than Grade III and no periventricular leukomalacia, were free of major congenital anomalies, and did not require surgery and whose parents consented within 48 hours of birth were included in the study. 

Enrolled infants were randomly assigned to the sucrose or water group from a computer-generated schedule. Only the project nurses in each site knew the group assignment; treating clinicians were blind to group assignment. Solutions of 0.1 mL of 24% sucrose or water were drawn up into sterile syringes and placed in the unit medicine refrigerator. Every time the infant was to undergo an invasive (e.g., heel lance, intravenous cannulation, arterial puncture, and injection) or noninvasive but presumably uncomfortable procedure (e.g., endotracheal tube suctioning, tape/lead removal, and gavage insertion for feeding), the solution in the syringe was administered into the infant's mouth 1 minute before the beginning of the procedure. 

A small-wide-angle lens camera rested on top of the isolette and was connected to a mat on the floor next to the isolette such that stepping on the mat triggered 5-minute recording. In this way, facial actions could be recorded during painful procedures. While the original study verified the analgesic effect of sucrose in minutes following the procedure, the current study evaluated the hedonic response using the first 15 seconds of facial capture after the sucrose solution was given orally and immediately before the painful procedure. We used filming from the very first time that the newborn received the oral solution, which occurred in the first 24 hours of life for all the subjects in the current study.

The positive hedonic reaction was compiled by adding scores for (a) rhythmic extension of the tongue outwards along the midline, and sometimes upwards, past the outer edge of the lips, often simultaneously accompanied by slight dropping of the jaw; this is followed immediately by retraction of the tongue and closure of the jaw, and the cycle is repeated rhythmically again and again, each cycle lasting 300–1200 ms and (b) lateral nonrhythmic tongue protrusions, which were sweeping extensions of the tongue sideways along the lateral border of the mouth and along the lips on one side of the mouth [15, 22]. A trained observer blinded to the solution given scored the videotapes frame-by-frame (1 frame = 1/10 s) watching the first 15 seconds of shooting after the administration of the oral solution. 

The definition of IUGR was based on the birth weight ratio (BWR), which is the ratio between the infant birth weight and the sex-specific mean birth weight for each gestational age for the local population [23, 24]. For this study, BWR was used as a continuous variable reflecting the degree of IUGR for a given infant.

### 2.1. Statistical Methods

Quantitative variables were described using median (25th percentile; 75th percentile), while categorical data were described using absolute (*n*) and relative (%) frequencies. To establish potential confounders, children who received sucrose or water were compared on key variables including gender, gestational age, birth weight, and BWR using Mann-Whitney test for quantitative variables and Fisher's Exact test for categorical variables.

Spearman correlation was used to analyze the relation between BWR and the hedonic response according to the solution offered. Statistical significance for all analyses was set at *P* < 0.05. 

## 3. Results

Table 1 depicts the baseline characteristics of subjects receiving the different solutions. There were no statistical differences between children that received water versus sucrose regarding gender, birth weight, gestational age, and birth weight ratio (Table 1).

**Figure 1:**
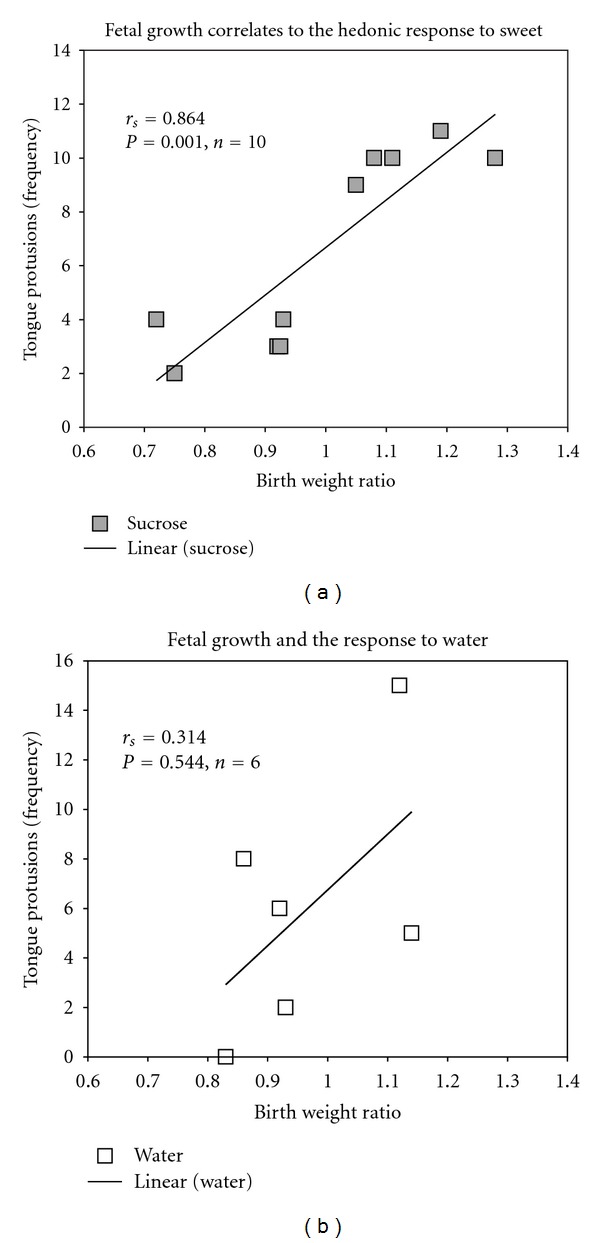
Correlations between birth weight ratio and the frequency of tongue protrusions (hedonic response) to (a) sucrose and (b) water.

There was a positive correlation between the BWR used as a continuous variable and the hedonic response to the sweet solution in the first 15 seconds after the offer (*r* = 0.864, *P* = 0.001). That is, the greater degree of intrauterine growth restriction, the less the frequency of the tongue protrusions demonstrated after the sucrose offer (Figure 1(a)). There was no correlation between BWR and the hedonic response when the solution given was water (*r* = 0.314, *P* = 0.544) (Figure 1(b)).

## 4. Discussion

In this study we showed that intrauterine growth correlates with the hedonic response to sweet in preterm infants in their first day of life. In preterm newborns born as early as 27 weeks gestation, the intensity of growth restriction is highly and inversely related to the frequency of positive affective reactions to the sweet taste. One could propose that IUGR leads to a decreased sensitivity to the enjoyment elicited by the sweet taste and would possibly overconsume this and other types of palatable foods when trying to reach a higher degree of pleasure. 

Interestingly, the current study agrees with our previous findings [10, 13] as well as with reports from other groups [11, 12] demonstrating that IUGR is indeed associated with an increased consumption of palatable foods at different times during the life-course. At age 24, women born severely growth restricted were shown to prefer to eat more carbohydrates and less protein than women born non-growth restricted, which was accompanied by an increased waist to hip ratio in this group [10]. Besides, people exposed to undernutrition during early fetal stages (the Dutch Famine birth cohort) were more likely to consume a high-fat diet at middle age and had more pronounced hypercholesterolemia, hypertriglyceridemia, and a twofold prevalence of coronary heart disease when compared to nonexposed individuals [11]. In another study involving a different sample of subjects exposed to the Dutch Famine in the first half of gestation, it was shown that they had higher reported absolute intakes of energy, fat, and protein and lower reported absolute intakes of carbohydrate than did the controls at middle age [12]. 

In addition, we recently showed that among children with normal birth weights, 3-year-old girls show a significantly greater ability to delay responding in a task using sweet as a reward when compared to boys. However, among IUGR girls, this ability to delay responding is lost. Furthermore, this impulsive responding at 36 months in girls predicts both increased consumption of palatable fat at 48 months of age and higher BMIs at 48 months of age [13]. Therefore, it seems that IUGR programs the hedonic response to palatable foods (such as sweet flavor), leading to an altered behavior when facing this type of foods that is seen already in childhood [13] and to an increased consumption in adult life [10–12]. The chronic increased ingestion of these aliments could, in the long term, lead to overweight, atherosclerosis, and cardiovascular disease already described in this group [4–9, 25, 26]. Alternatively, the altered response to the sweet taste in a critical period by itself programs the neural circuits responsible for the establishment of food preferences, leading to altered feeding choices in adult life in IUGR individuals.

Considering that the taste reactivity facial reactions are homologous in humans and animals [15, 16], and therefore share underlying brain circuits, one could argue that IUGR programs the functioning of these circuits during fetal life. Berridge and coworkers have described “hedonic hotspots” in the nucleus accumbens and ventral pallidum that use opioid and endocannabinoid signals to amplify the hedonic response for sweetness [27]. Microinjection of the opioid agonist DAMGO in these areas in rats causes sucrose taste to elicit over twice as many hedonic reactions as it normally does [27–30]. Similarly, microinjection of anandamide, which likely acts by stimulating the CB1 type of cannabinoid receptor, doubles the number of positive hedonic facial reactions that sucrose taste elicits from rats [29, 31]. Intrauterine growth restriction may decrease the sensitivity of these systems, by diminishing the action of the mu subtype of opioid receptor or the CB1 type of cannabinoid receptor, leading to a decrease in the hedonic response to the sweet taste.

The apparent contradiction of less hedonic response early in life versus increased consumption in adulthood could be explained by a detachment of the normal close association between the hedonic value (or “liking”) and the incentive salience (or “wanting”). This could lead to motivated food consumption that is no longer hedonically driven by the activation of mesolimbic dopamine mechanisms of incentive salience, or even opioid circuits outside the hedonic hotspots. The suppression of positive hedonic reward systems or activation of dysphoric stress systems might prompt persistent attempts to use palatable food as a relief [32].

Our study has some limitations. Mainly, the small number of participants did not allow us to employ more adequate statistical adjustments (e.g., considering SES and gender in the analysis) and to classify the newborns into IUGR or non-IUGR categories. In addition, other components of the hedonic reaction could not be scored for the angle of the shooting performed. However, the correlation is very intense for a biological variable, especially considering that the study outline and the selection of participants were not primarily designed to test the current hypothesis.

In conclusion, this is the first evidence of fetal programming of the hedonic response to the sweet taste in humans by IUGR. This takes on added significance considering that these individuals also show a persistent preference for palatable foods later in life [10–12], as well as an increased risk for overweight and related metabolic and cardiovascular consequences [4–9, 25, 26]. Such group of evidence [33] could potentially bring enlightment to future studies aiming primary prevention measures in this population. 

## Figures and Tables

**Table 1:** Study participants' baseline characteristics according to the solution given.

Sample characteristics	Sucrose (*n* = 10)	Water (*n* = 6)	*P*
Males (%)	6 (60%)	4 (66.6%)	0.61^§^
Birth weight (g)	1032.50 (810.00; 1320.00)	975.00 (822.50; 1411.25)	0.91^∗^
Gestational age (weeks)	28.00 (25.00; 29.0)	27.50 (26.50; 29.00)	0.87^∗^
Birth weight ratio	0.99 (0.88; 1.13)	0.92 (0.85; 1.12)	0.75^∗^

^∗^Mann-Whitney test and ^§^Fisher's Exact test. Data are expressed as median (25th percentile; 75th percentile) or *n* (percentages).
